# Subjective Assessment of Head and Facial Appearance in Children with Craniosynostoses after Surgical Treatment

**DOI:** 10.3390/healthcare6040127

**Published:** 2018-10-24

**Authors:** Dawid Larysz, Elżbieta Nieroba

**Affiliations:** 1 Department of Radiotherapy, Maria Skłodowska-Curie Memorial Cancer Center and Institute of Oncology, Gliwice Branch, Wybrzeże AK 15, 44-101 Gliwice, Poland; dawilar@gmail.com; 2 Institute of Sociology, University of Opole, Katowicka 89, 45-061 Opole, Poland

**Keywords:** craniosinostosis, results of surgery, craniofacial disfigurement, clinical survey, subjective assessment, surgical outcomes

## Abstract

Background: Craniosynostoses are congenital defects in the construction of the skull involving premature fusion of one or more cranial sutures. Premature fusion of sutures causes characteristic skull deformation(s). This affect the structure and thus the appearance of the entire head and face. The aim of this study was to analyze parents’ subjective assessments of head and facial appearance in children with craniosynostoses before and after surgery. Parents also assessed the interpersonal relationship of their children with peers and adults (after surgery). Methods: This study was conducted among parents of 230 children treated in Poland, in two multidisciplinary centers. Detailed statistical analysis was conducted among children who had undergone surgery. Independent variables were age (at survey) of the child (three years and less, four years, and five years and more) and type of craniosynostosis (isolated and syndromic). A chi-square independence test was used. Data was collected using surveys. Results: In the opinion of most parents, the appearance of their child’s head and face after surgery did not differ or differed only slightly from that of their peers. The results of subjective assessment of appearance of children’s face and head after reconstructive treatment remains comparable in three subgroups of patients according to the age. It seems that specific head shape according to the type of craniosynostosis does not have an impact on relations with peers and adults. Conclusion: Surgical treatment of children with craniosynostoses improves the appearance of their head and face. This improvement seems not to depend on the type of isolated craniosynostosis, and is constant over time.

## 1. Introduction

Especially important to identity is the face, which in contrast to the rest of the body is always exposed [1]. This means that “the face tells others about who we are” [2]. The face plays a key role in building and maintaining social relations. An atypical or distorted face may cause problems in social interactions and affect individual self-esteem [3,4,5].

Numerous studies show that craniofacial disfigurement affects various aspects of the lives of those suffering therefrom as well as their loved ones. Parents of children with craniofacial anomalies may fear the reactions of others to the appearance of their child, and wonder whether their child will be accepted. Furthermore, parents may find it difficult to talk frankly with their child about his or her craniofacial differences, and/or become overprotective [6] (pp. 93, 98). Children with craniofacial disfigurement are more prone to negative self-perception and low self-esteem [6] (pp. 93–94) [7]. Adolescents with craniofacial anomalies feel less attractive, and are ridiculed by their peers [8,9,10,11,12].

Facial disfiguration can be congenital or acquired [6]. Acquired disfigurements can be caused by injury, disease, or surgical treatment (e.g., scars). One example of congenital malformation of the entire head and the face is craniosynostoses.

Craniosynostoses are a heterogenous group of congenital diseases occurring in one out of every 2000–2500 live births. Although they are the second most common group of facial and skull defects after orofacial clefts, their frequency classifies them as a rare disease [13,14].

The surgical treatment of children with craniosynostoses is based on multidisciplinary reconstruction of the skull, including reconstruction of the upper and/or middle part of the face.

The aim of this study was to analyze parents’ subjective assessments of the appearance of the head and face of children with craniosynostoses. The interpersonal relationships of these children were also assessed. More specifically, we will focus on: (1) if there are any statistically significant differences in subjective assessment of the child’s head and face appearance before and after surgical correction; (2) if specific etiology of craniosynostosis (syndromic vs. non-syndromic) is responsible for objective assessment of the child’s head and face; (3) if the age of the child influences the assessment of the head and face appearance; and (4) if the children with craniosynostoses after head reconstruction had problems with social relations with peers and adults.

## 2. Methods

### 2.1. Participants and Research Strategies

The study was conducted among parents of children with various types of craniosynostoses treated in Poland, in two multidisciplinary centers: the John Paul II Upper Silesian Child Health Centre in Katowice; and the Craniofacial Treatment Centre in Olsztyn. Over 60% of Polish children with craniosynostoses are treated in these centers. The sample group consisted of 230 children with various craniosynostoses. Inclusion criteria consisted of diagnosis of craniosynostosis made by pediatric neurosurgeon according to clinical examination and 3D-CT scan. We exclude children with any other than non-syndromic or syndromic craniosynostosis abnormalities.

Data was collected using surveys. Surveys were conducted during control visits in craniofacial centers. Parents were asked to complete the questionnaire anonymously. The questionnaire (see Appendix A) consisted of three sections, concerning: (1) Basic child data—gender, age at the time of survey (the children were divided into three subgroups according to age: three years and less; four years; and five years and more), type of craniosynostosis (sagittal, metopic, unicoronal, bicoronal, lambdoid, complex, genetic syndrome). The parents of children who had undergone surgery were also asked to assess: (2) the head and facial appearance of their children before and after surgery and (3) child’s interpersonal relationship after surgery. The response rate was 91%.

### 2.2. Data Analysis

Statistical analysis was performed using IMAGO Academic (vendor—Predictive Solutions, Kraków, Poland). A chi-square independence test (*p* < 0.05) was used to test the relationships between the following variables: parent assessment of child’s head appearance before and after surgery; parent assessment of child’s facial appearance before and after surgery; type of craniosynostosis (isolated and syndromic) and assessment of child’s head appearance before and after surgery; type of craniosynostosis (isolated and syndromic) and assessment of child’s face appearance before and after surgery; type of craniosynostosis (isolated and syndromic) and reactions of peers on children’s head (after surgery); type of craniosynostosis (isolated and syndromic) and reactions of adults on children’s head (after surgery); the children’s age and assessment of their head appearance after surgery; the children’s age and assessment of their face appearance after surgery; the children’s age and their relations with peers (after surgery); and the children’s age and their relations with adults (after surgery).

## 3. Results

### 3.1. Demographics

Boys constituted the majority of the sample (157/230 or 68.2%). The average age at the time of survey was 42.68 months (SD 30.18, min. 4, max. 159). The most common types were sagittal craniosynostosis (96/230 or 41.8%) and metopic craniosynostosis (80/230 or 34.7%). 211/230 or 91.7% of the children had undergone skull reconstruction surgery. Their average age at the time of surgery was 13.26 months (SD 8.69, min. 2, max. 71).

### 3.2. Parent Assessment of Head and Facial Appearance before and after Surgery

Seventy-six percent of parents claimed that the appearance of their child’s head after surgery did not differ or differed only slightly from that of their peers. 58% claimed that before surgery, the irregular head was noticeable by all or most people. Analysis showed a statistically significant difference between the before and after (*p* = 0.015).

**Figure 1:**
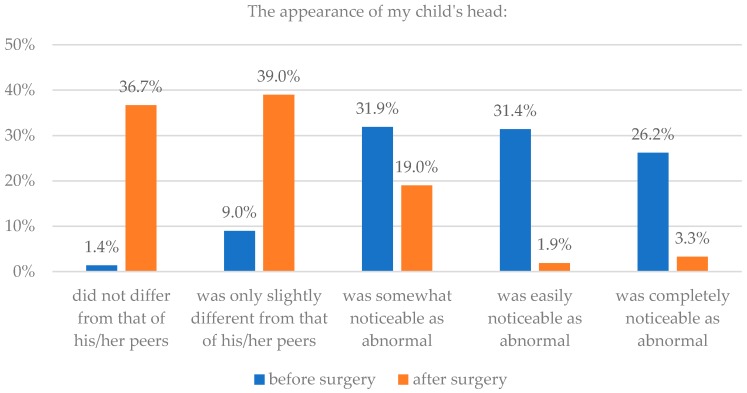
Assessment of head appearance before and after surgery.

**Figure 2:**
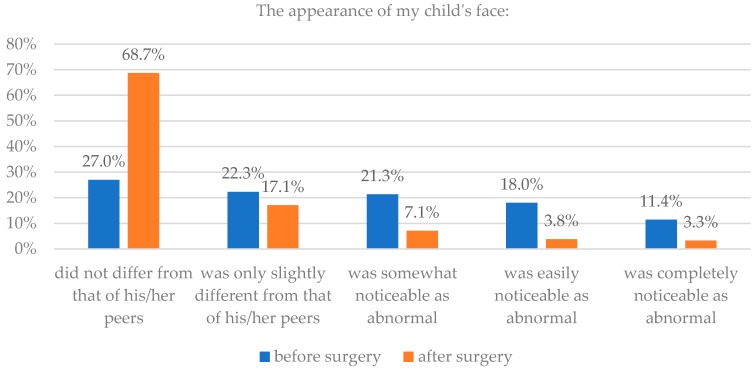
Assessment of facial appearance before and after surgery.

Parents also saw an improvement in the appearance of their child’s face after operation. 86% parents thought that their child’s face did not differ or differed only slightly from that of their child’s peers after surgery, while only 49% thought so before surgery. Assessments of appearance before and after surgery were statistically significantly different for the entire sample group (*p* < 0.0001). Details are presented in Table 1 and Figure 1 and Figure 2.

### 3.3. Influence of Type of Craniosynostosis on Parents’ Assessment of Head and Facial Appearance before and after Surgery

**Figure 3:**
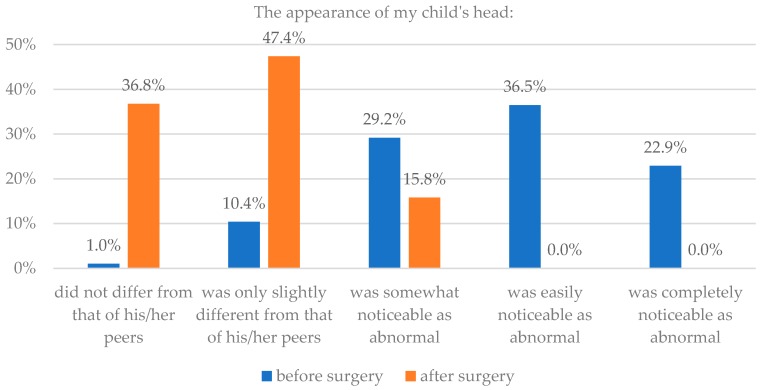
Isolated craniosynostoses and assessment of head appearance before and after surgery.

**Figure 4:**
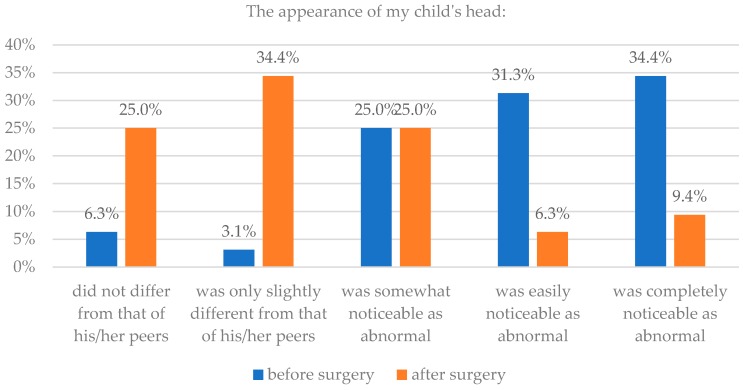
Syndromic craniosynostoses and assessment of head appearance before and after surgery.

Based on clinical knowledge of 128 parents the sample was divided into two groups—those with isolated craniosynostoses and those with genetic defects (96/128 or 75%, and 32/128 or 25%, respectively). It was revealed that if head appearance before and after surgery was significantly different in case of children without mutations (*p* = 0.016), while it was not significantly different before and after surgery in case of children with genetic syndromes (*p* = 0.18). Details are presented in Table 2 and Figure 3 and Figure 4.

**Figure 5:**
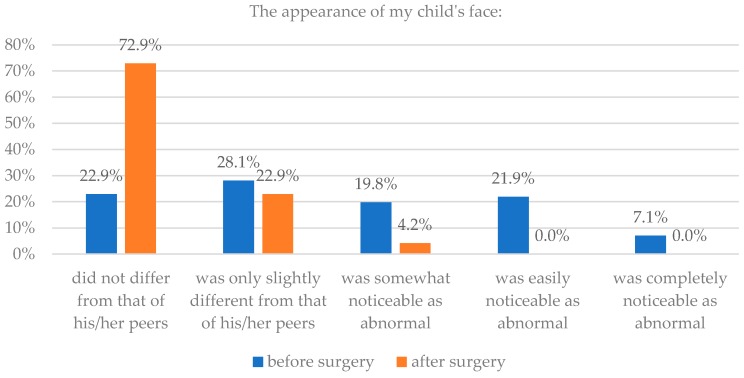
Isolated craniosynostoses and assessment of face appearance before and after surgery.

**Figure 6:**
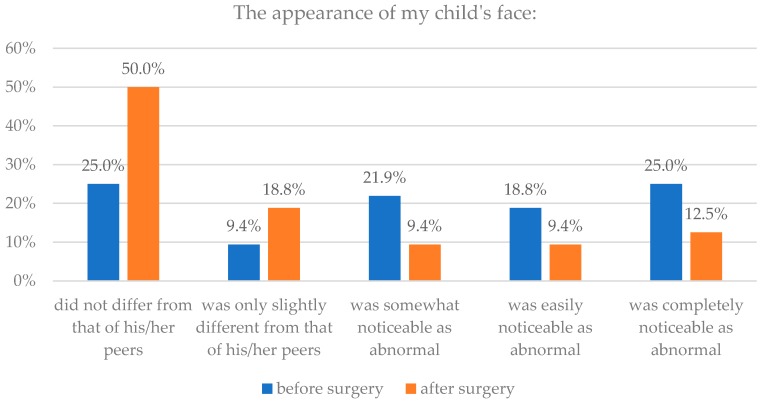
Syndromic craniosynostoses and assessment of face appearance before and after surgery.

The analysis revealed also statistically significant differences in facial assessment before and after surgery in subgroup of children with syndromic craniosynostoses (*p* = 0.005), on the other hand we did not find significant differences in subgroup with non-syndromic craniosynostoses (*p* = 0.075). Details are presented in Table 3 and Figure 5 and Figure 6.

### 3.4. The Children’s Age (at the Moment of Survey) and Assessment of Head and Facial Appearance after Surgery

**Figure 7:**
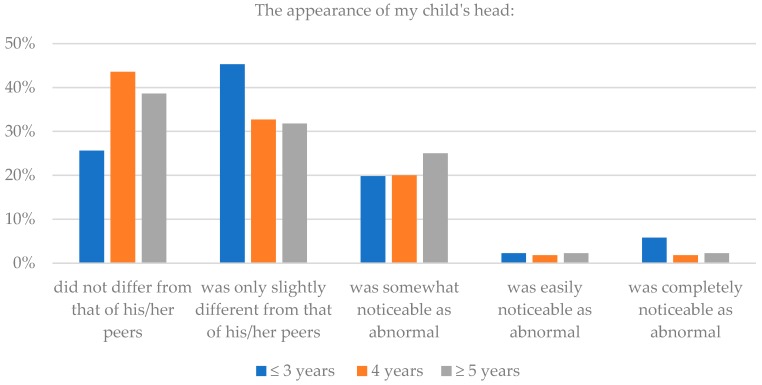
The children’s age and assessment of head after surgery.

**Figure 8:**
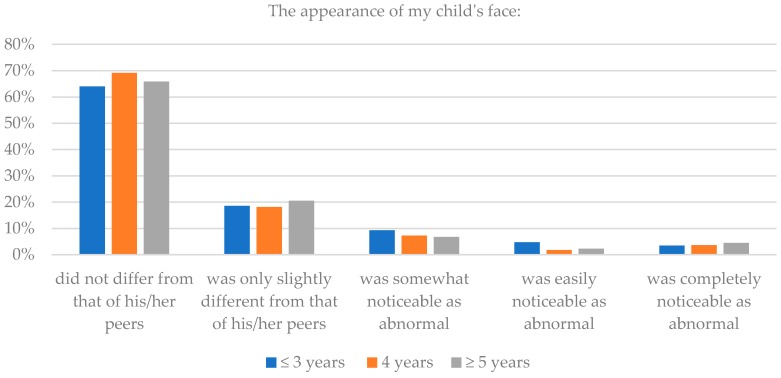
The children’s age and assessment of face after surgery.

Seventy percent of parents of children three years of age and less thought that after surgery the appearance of their child’s head did not differ or differed only slightly from that of his/her peers, while 8.1% thought that it differed greatly. Eighty-three percent of parents thought that the appearance of their child’s face did not differ or differed only slightly from that of his/her peers, while 8.2% thought that it differed greatly. For parents of children four years, similar positive assessment were 76.3% and 87.3%, respectively for the head and the face assessment; and for parents of children 5 years and more, 70.4% and 86.4%, respectively. Additionally, in this case, there were no statistically significant differences within these subgroups (*p* = 0.145), which again suggests a consistent aesthetic effect of reconstruction surgery. Details are presented in Table 4 and Figure 7 and Figure 8.

### 3.5. Parents’ Assessment of Their Child’s Interpersonal Relationships after Surgery

Parents do not view their children had problems relating to other children (86.2%), or adults (86.2%) (details are presented in Table 5). They also declared that their children “really like” or “like” to play with peers (64.6% and 27.3%, respectively). Only 2.4% of parents declared that their child “really does not like” or “does not like” to play with peers. Likewise, 85% of parents declared that their child’s peers “really like” or “like” to play with their child (28.8% and 56.3%, respectively) (data not presented in the table).

Within the individual age groups, there were no statistically significant differences in relations between peers and adults. However, there was a statistically significant reduction in the frequency of negative reactions of both peers (*p* = 0.014) and adults (*p* = 0.001) to the head shape of children with syndromic craniosynostoses after such treatment. This was not true in case of willingness to play with and relate to peers and adults. The type of isolated craniosynostosis did not seem to have an impact on relations with peers or adults, or their reactions to head shape.

## 4. Discussion

The main force that is responsible for head growth of fetuses and infants is the brain as it rapidly expands inside the skull cavity. Cranial sutures are fibrous joints between the cranial vault and the skull base, and they are one of the key factors that allows head growth. Craniosynostoses are congenital defects in the construction of the skull involving premature fusion of one or more cranial sutures. Premature fusion of sutures causes characteristic skull deformation depending on which suture is fused. The most common types of craniosynostoses are: sagittal synostosis (premature fusion of the sagittal suture); metopic synostosis (fusion of the metopic suture); unicoronal synostosis (unilateral or bilateral fusion of the coronal suture); bicoronal synostosis (simultaneous fusion of the sagittal and metopic suture); and complex synostosis (simultaneous fusion of two or more sutures).

Etiology of craniosynostoses is diverse and in most cases still not clear. It is estimated that around 90% of craniosynostoses cases are isolated and non-syndromic. The remaining 10% of cases occur as part of syndromes with genetic etiology. The most common are mutations in *FGRF1*, *FGRF2*, *FGRF3*, or *TWIST1* genes. The most important syndromes are Crouson syndrome, Apert syndrome, Muenke syndrome, Pfeiffer syndrome, and Saethre-Chotznen syndrome. However, recent research indicates that some defects clinically classified as non-syndromic also involve genetic mutations [15].

We should emphasize that the premature fusion of the single suture causes abnormal shape of entire head, even when there are only small fused sutures around the base of the skull. Such abnormal shape and structure of the skull vault always affects also facial structure. The deformation pattern depends on the type of craniosynostosis. The most common abnormalities in the face are problems with positioning of the orbits causing hipotelorism, hypertelorism, and/or dystopia. Premature closure of unilateral coronal suture usually lead to more or less severe facial scoliosis. In children with syndromic symptoms, the craniosynostosis does not only affect the head and face, but also could affect other bones and organs over the entire body. In some syndromes head deformity can also occur concomitantly with a cleft lip or palate. Another characteristic concomitant deformation is polisyndactylia of the feet and hands and/or other limbs abnormalities concerning bones and joints. The parents of patients, as well as patients themselves, often indicate that, in addition to their physical consequences, the above deformities also have psychological and social consequences [16,17,18].

There is much controversy over the specific goals of craniosynostosis treatment in children. The indications that treatment reduces intracranial hypertension and corrects significant bone deformities that affect bodily functions such as breathing, food intake, eyesight, etc., are obvious and indisputable [19,20]. Yet there is a fairly large number of children with isolated craniosynostosis who do not suffer from intracranial hypertension. The speech impediments, muscle tension, and even vision impairment occurring various percentage of children according to type of craniosynostosis and can be treated conservatively through speech therapy, physical therapy, and multi-profile child development support [21,22,23,24,25]. This poses the question of whether it is reasonable to take the risks associated with reconstruction surgery.

Aesthetic appearance of the head and the face are also important factors in qualification process, preoperative planning of surgery and assessment of the surgical results not only from medical but also psychological and social points of view [26,27,28,29].

The aim of our study was assessment of surgical results in context of head and face appearance and their influence on interpersonal relationship.

Clinical assessment of craniosynostosis correction is still a point of debate. The most common is assessment by craniofacial specialist done according to Whitaker classification system that is simple and widely used [29,30]. The classification system were categorized on the basis of the need for additional surgery for correction of still visible aesthetical abnormalities. In some studies the aesthetic and clinical results after surgical treatment remain stable [31]. Unfortunately other authors demonstrate a clear trend toward worsening aesthetic outcomes over time [29]. On the other hand the authors in the next research showed that the Whitaker classification exhibits low interrater reliability and could not predict future treatment [29]. That is why subjective non specialist-specific assessment is needed for treatment results evaluation. Care et al. [28] developed a photobook consisted of preoperative and postoperative photographs of patients. They showed that such method could be helpful for parents to make decision about surgery and for proper understanding of aesthetic surgical results [28]. There are no studies in Polish medical literature concerning subjective assessment of surgical results of children with craniosynostoses.

In our research the majority of parents declared that after surgery, the appearance of their child’s head and face (76% and 86%, respectively) did not differ from that of their peers. Furthermore, most parents declared that their children did not have problems relating to other adults (87.6%) or peers (86.2%), and that they were not ignored by their peers (85%). One important revelation from this study was that the positive aesthetic and social effects of surgery are stable over time (in children 3, 4 and 5+ years old). Further studies of adolescent children would definitely provide more information on this clinically important subject [32]. Salokorpi et al. [33] analyzed the satisfaction with facial appearance and attractiveness of 40 adults who as children had undergone cranial reconstruction due to non-syndromic sagittal synostosis, as rated by two independent panels. The study revealed that patients who had been treated surgically were as happy with their facial appearance as individuals in an age- and gender-matched control group. They evaluated their appearance as only somewhat less attractive.

Another important revelation is as follows. Children with craniosynostoses often have neurodevelopmental disorders, which affect how they function in both school and family environments. Symptoms include clumsiness, problems in school, vision impairment, and speech impairment [22,34,35]. Each of these alone is often enough to reduce self-esteem and quality of life. Such symptoms are usually more pronounced in children who have not undergone reconstructive surgery, and further hinder peer contact and social function. These strictly subjective consequences are very rarely considered as indications for surgery. This research reveals that skull reconstruction surgery in early childhood improves head and facial appearance, as well as social function. Due to these accompanying disorders and more serious, often progressive deformities, children with genetic defects benefit less from surgery (both clinically and socially) than children with isolated craniosynostoses. Nevertheless, this study shows that surgery does improve the appearance and social function of children with genetic defects.

Almost all respondents (97.7%) declared that they would recommend surgery to other parents of children with craniosynostoses.

### Limitations

One of major limitations of this survey is that it is conducted after surgery, not both before and after surgery. The bias may thus be introduced based upon our clinical experience. This study analyzed the opinions of parents of children with craniosynostoses. Due to the age of the children, the children themselves were unable to assess the appearance of their head and face, or their interpersonal relations. We are planning to study this in the future, and correlate the results with the assessments of their parents. The size and age distribution of the analyzed group did not allow for comparison of children who had undergone surgery with those who had not (there were only a few such children, and they were very young). Finally, separate analysis of children with genetic defects as part of syndromes may provide more specific insight into this extremely heterogenous group.

## 5. Conclusions

Our survey suggests that: (1) Operative reconstructive treatment of children with craniosynostoses seems to improve the appearance of their head and face; (2) this improvement seems not to depend on the type of isolated craniosynostosis, and is constant over time; (3) such treatment is less effective in children with genetic craniosynostoses than in those with isolated craniosynostoses; and (4) all parents in this study declared that their children’s relations with both peers and the rest of society did not differ from those of their unafflicted peers.

## Figures and Tables

**Table 1:** Assessment of head and facial appearance before and after surgery.

Parents’ Assessment	The Appearance of My Child’s Head				The Appearance of My Child’s Face			
	Before Surgery		After Surgery		Before Surgery		After Surgery	
	n	%	n	%	n	%	n	%
Did not differ from that of his/her peers	3	1.4	77	36.7	57	27.0	145	68.7
Was only slightly different from that of his/her peers	19	9.0	82	39.0	47	22.3	36	17.1
Was somewhat noticeable as abnormal	67	31.9	40	19.0	45	21.3	15	7.1
Was easily noticeable as abnormal	66	31.4	4	1.9	38	18.0	8	3.8
Was completely noticeable as abnormal	55	26.2	7	3.3	24	11.4	7	3.3

**Table 2:** Type of craniosynostosis and assessment of head appearance before and after surgery.

The Appearance of My Child’s Head								
Parents’ Assessment	Isolated Craniosynostoses				Syndromic Craniosynostoses			
	Before Surgery		After Surgery		Before Surgery		After Surgery	
	n	%	n	%	n	%	n	%
Did not differ from that of his/her peers	1	1.0	35	36.8	2	6.3	8	25.0
Was only slightly different from that of his/her peers	10	10.4	45	47.4	1	3.1	11	34.4
Was somewhat noticeable as abnormal	28	29.2	15	15.8	8	25.0	8	25.0
Was easily noticeable as abnormal	35	36.5	0	0.0	10	31.3	2	6.3
Was completely noticeable as abnormal	22	22.9	0	0.0	11	34.4	3	9.4

**Table 3:** Type of craniosynostosis and assessment of face appearance before and after surgery.

The Appearance of My Child’s Face								
Parents’ Assessment	Isolated Craniosynostoses				Syndromic Craniosynostoses			
	Before Surgery		After Surgery		Before Surgery		After Surgery	
	n	%	n	%	n	%	n	%
Did not differ from that of his/her peers	22	22.9	70	72.9	8	25.0	16	50.0
Was only slightly different from that of his/her peers	27	28.1	22	22.9	3	9.4	6	18.8
Was somewhat noticeable as abnormal	19	19.8	4	4.2	7	21.9	3	9.4
Was easily noticeable as abnormal	21	21.9	0	0.0	6	18.8	3	9.4
Was completely noticeable as abnormal	7	7.1	0	0.0	8	25.0	4	12.5

**Table 4:** The children’s age and assessment of head and face appearance after surgery.

Parents’ Assessment	The Appearance of My Child’s Head			The Appearance of My Child’s Face		
	≤3 Years (%)	4 Years (%)	≥5 Years (%)	≤3 Years (%)	4 Years (%)	≥5 Years (%)
Did not differ from that of his/her peers	25.6	43.6	38.6	64.0	69.1	65.9
Was only slightly different from that of his/her peers	45.3	32.7	31.8	18.6	18.2	20.5
Was somewhat noticeable as abnormal	19.8	20.0	25.0	9.3	7.3	6.8
Was easily noticeable as abnormal	2.3	1.8	2.3	4.7	1.8	2.3
Was completely noticeable as abnormal	5.8	1.8	2.3	3.5	3.6	4.5

**Table 5:** Parents’ assessment of their child’s interpersonal relationships (after surgery).

Parents’ Assessment	How Does Your Child Relate to Peers		How Does Your Child Relate to Adults	
	n	%	n	%
Very easily	87	41.4	70	33.3
Rather easily	94	44.8	114	54.3
Hard to say	19	9.0	17	8.1
Not very well	6	2.9	7	3.3
Poorly	4	1.9	2	1.0

## Appendix A

**Questionnaire:**

- Child’s sex: girl/boy
- Age of child (in months)
- Type of craniosynostosis: sagittal; metopic; unicoronal; bicoronal; lambdoid; complex; genetic syndrome
- Surgery: yes/no

Questions for parents with child after surgery

- Age of child at the time of surgery (in months)
- The appearance of my child’s head before surgery: did not differ from that of his/her peers; was only slightly different from that of his/her peers; was somewhat noticeable as abnormal; was easily noticeable as abnormal; was completely noticeable as abnormal
- The appearance of my child’s head after surgery: do not differ from that of his/her peers; is only slightly different from that of his/her peers; is somewhat noticeable as abnormal; is easily noticeable as abnormal; is completely noticeable as abnormal
- The appearance of my child’s face before surgery: did not differ from that of his/her peers; was only slightly different from that of his/her peers; was somewhat noticeable as abnormal; was easily noticeable as abnormal; was completely noticeable as abnormal
- The appearance of my child’s face after surgery: do not differ from that of his/her peers; is only slightly different from that of his/her peers; is somewhat noticeable as abnormal; Is easily noticeable as abnormal; is completely noticeable as abnormal
- How does your child relate to peers after surgery: very easily; rather easily; hard to say; not very well; poorly
- How does your child relate to adults after surgery: very easily; rather easily; hard to say; not very well; poorly
- Whether peers react negatively to the shape of your child’s head after surgery: never; seldom; sometimes; often; always
- Whether adults react negatively to the shape of your child’s head after surgery: never; seldom; sometimes; often; always
- Does your child like to play with peers after surgery: really like; like; hard to say; does not like; really does not like
- How do peers relate to your child after surgery: very easily; rather easily; hard to say; not very well; poorly

## References

[1] Kępiński A. (1997). Twarz i ręka. Teksty Teor. Lit. Kryt. Interpret..

[2] Talley H.L. (2014). Saving Face. Disfigurement and the Politics of Appearance.

[3] Goffman E. (1963). Behavior in Public Places. Notes on the Social Organization of Gatherings.

[4] Goffman E. (1967). Interaction Ritual. Essays in Face-to-Face Behawior.

[5] Macgregor F.C. (1990). Facial disfigurement: Problems and management of social interaction and implications for mental health. Aesthet. Plast. Surg..

[6] Rumsey N., Hacourt D. (2005). The Psychology of Appearance.

[7] Pope A.W., Ward J. (1997). Self-perceived facial appearance and psychosocial adjustment in preadolescents with craniofacial anomalies. Cleft Palate Craniofac. J..

[8] Prior J., O’Dell L. (2009). ‘Coping quite well with a few difficult bits’ living with disfigurement in early adolescence. J. Health Psychol..

[9] Kish V., Lansdown R. (2000). Meeting the psychosocial impact of facial disfigurement: Developing a clinical service for children and families. Clin. Child Psychol. Psychiatry.

[10] Caroll P., Shute R. (2005). School peer victimization of young people with craniofacial conditions: A comparative study. Psychol. Health Med..

[11] Harlock N., Vögelin E., Bradbury E.T., Grobbelaar A.O., Gault D.T. (2005). Psychosocial outcome of patients after ear reconstruction: A retrospective study of 62 patients. Ann. Plast. Surg..

[12] Kapp-Simon K.A., McGuire D. (1997). Observed social interaction patterns in adolescents with and without craniofacial conditions. Cleft Palate Craniofac. J..

[13] Lajeunie E., Le Merrer M., Marchac D., Renier D. (1998). Syndromal and nonsyndromal primary trigonocephaly: Analysis of a series of 237 patients. Am. J. Med. Genet..

[14] Slater B.J., Lenton K.A., Kwan M.D., Gupta D.M., Wan D.C., Longaker M.T. (2008). Cranial sutures: A brief review. Plast. Reconstr. Surg..

[15] Timberlake A.T., Furey C.G., Choi J., Nelson-Williams C., Loring E., Galm A., Kahle K.T., Steinbacher D.M., Larysz D., Persing J.A. (2017). De novo mutations in inhibitors of Wnt, BMP, and Ras/ERK signaling pathways in non-syndromic midline craniosynostosis. Proc. Natl. Acad. Sci. USA.

[16] Kana M.A., Baduku T.S., Bello-Manga H., Baduku A.S. (2018). A 37-year-old Nigerian woman with Apert syndrome—Medical and psychosocial perspectives: A case report. J. Med. Case Rep..

[17] Lloyd M.S., Venugopal A., Horton J., Rodrigues D., Nishikawa H., White N., Solanki G., Noons P., Evans M., Dover S. (2016). The quality of life in adult patients with syndromic craniosynostosis from their perspective. J. Craniofac. Surg..

[18] Sandy R., Hennocq Q., Nysjö J., Giran G., Friess M., Khonsari R.H. (2018). Orbital shape in intentional skull deformations and adult sagittal craniosynostoses. J. Anat..

[19] Wall S.A., Thomas G.P., Johnson D., Byren J.C., Jayamohan J., Magdum S.A., McAuley D.J., Richards P.G. (2014). The preoperative incidence of raised intracranial pressure in nonsyndromic sagittal craniosynostosis is underestimated in the literature. J. Neurosurg. Pediatr..

[20] Thomas G.P., Johnson D., Byren J.C., Judge A.D., Jayamohan J., Magdum S.A., Richards P.G., Wall S.A. (2015). The incidence of raised intracranial pressure in nonsyndromic sagittal craniosynostosis following primary surgery. J. Neurosurg. Pediatr..

[21] Larysz D., Rożek A. (2016). Incorrect structure of articulation organs and delayed speech development in children with non-syndromic craniosynostoses. Logop. Silesiana.

[22] Larysz D. (2012). Disorders of speech development in children with deformity of the skull. Logop. Silesiana.

[23] Naran S., Miller M., Shakir S., Ware B., Camison L., Ford M., Goldstein J., Losee J.E. (2017). Nonsyndromic craniosynostosis and associated abnormal speech and language development. Plast. Reconstr. Surg..

[24] Chuang C., Rolison M., Yang J.F., Brooks E.D., Hashim P.W., Travieso R., Terner J., Steinbacher D.M., Landi N., Stavropoulos K.K.M. (2018). Normalization of speech processing after whole-vault cranioplasty in sagittal synostosis. J. Craniofac. Surg..

[25] McCarthy J.G., Warren S.M., Bernstein J., Burnett W., Cunningham M.L., Edmond J.C., Figueroa A.A., Kapp-Simon K.A., Labow B.I., Peterson-Falzone S.J. (2012). Craniosynostosis Working Group. Parameters of care for craniosynostosis. Cleft Palate Craniofac. J..

[26] Taylor J.A., Paliga J.T., Wes A.M., Tahiri Y., Goldstein J.A., Whitaker L.A., Bartlett S.P. (2015). A critical evaluation of long-term aesthetic outcomes of fronto-orbital advancement and cranial vault remodeling in nonsyndromic unicoronal craniosynostosis. Plast. Reconstr. Surg..

[27] Martini M., Klausing A., Messing-Jünger M., Lüchters G. (2017). The self-defining axis of symmetry: A new method to determine optimal symmetry and its application and limitation in craniofacial surgery. J. Craniomaxillofac. Surg..

[28] Care H., Dalton L., Johnson D. (2018). The value of a photobook in informing families about the cosmetic results of surgery in craniosynostosis. J. Craniofac. Surg..

[29] Wes A.M., Paliga J.T., Goldstein J.A., Whitaker L.A., Bartlett S.P., Taylor J.A. (2014). An evaluation of complications, revisions, and long-term aesthetic outcomes in nonsyndromic metopic craniosynostosis. Plast. Reconstr. Surg..

[30] Whitaker L.A., Bartlett S.P., Schut L., Bruce D. (1987). Craniosynostosis: An analysis of the timing, treatment, and complications in 164 consecutive patients. Plast. Reconstr. Surg..

[31] Kreppel M., Kauke M., Safi A.F., Grandoch A., Pocek-Behn N., Nickenig H.J., Zöller J. (2018). Clinical evaluation of non-syndromic scaphocephaly surgically corrected with the procedure of total vertex craniectomy. J. Craniomaxillofac. Surg..

[32] Burokas L. (2013). Craniosynostosis: Caring for infants and their families. Crit. Care Nurse.

[33] Salokorpi N., Savolainen T., Sinikumpu J.J., Ylikontiola L., Sándor G.K., Pirttiniemi P., Serlo W. (2018). Outcomes of 40 nonsyndromic sagittal craniosynostosis patients as adults: A case-control study with 26 years of postoperative follow-up. Oper. Neurosurg..

[34] Wallace E.R., Collett B.R., Kapp-Simon K., Starr J.R., Birgfeld C., Speltz M.L. (2016). Visuomotor function in school-age children with single-suture craniosynostosis. J. Dev. Behav. Pediatr..

[35] Speltz M.L., Collett B.R., Wallace E.R., Kapp-Simon K. (2016). Behavioral adjustment of school-age children with and without single-suture craniosynostosis. Plast. Reconstr. Surg..

